# Optimization of SAW Sensors for Nanoplastics and Grapevine Virus Detection

**DOI:** 10.3390/bios13020197

**Published:** 2023-01-28

**Authors:** Silvia Rizzato, Anna Grazia Monteduro, Ilaria Buja, Claudio Maruccio, Erika Sabella, Luigi De Bellis, Andrea Luvisi, Giuseppe Maruccio

**Affiliations:** 1 Omnics Research Group, Department of Mathematics and Physics University of Salento, CNR-Institute of Nanotechnology, INFN Sezione di Lecce, Via per Monteroni, 73100 Lecce, Italy; 2 Department of Biological and Environmental Sciences and Technologies, University of Salento, Via Monteroni, 73100 Lecce, Italy

**Keywords:** surface acoustic waves, biosensors, lithium niobate, microplastics, particulate matter, plant pathogens, environmental monitoring

## Abstract

In this work, we report the parametric optimization of surface acoustic wave (SAW) delay lines on Lithium niobate for environmental monitoring applications. First, we show that the device performance can be improved by acting opportunely on geometrical design parameters of the interdigital transducers such as the number of finger pairs, the finger overlap length and the distance between the emitter and the receiver. Then, the best-performing configuration is employed to realize SAW sensors. As aerosol particulate matter (PM) is a major threat, we first demonstrate a capability for the detection of polystyrene particles simulating nanoparticulates/nanoplastics, and achieve a limit of detection (LOD) of 0.3 ng, beyond the present state-of-the-art. Next, the SAW sensors were used for the first time to implement diagnostic tools able to detect Grapevine leafroll-associated virus 3 (GLRaV-3), one of the most widespread viruses in wine-growing areas, outperforming electrochemical impedance sensors thanks to a five-times better LOD. These two proofs of concept demonstrate the ability of miniaturized SAW sensors for carrying out on-field monitoring campaigns and their potential to replace the presently used heavy and expensive laboratory instrumentation.

## 1. Introduction

The availability of miniaturized sensors with increasingly better performance and integration capabilities is having a revolutionary impact as key enabling technologies to address societal challenges and industrial needs. Their diffusion has promoted the development of new technology sectors and applications: from home automation to smart cities [1,2,3,4,5], from biomedical diagnostics to drug research [6,7,8,9,10,11] and from industrial and environmental monitoring [12,13,14,15] to precision agriculture [16,17,18,19,20,21,22,23,24,25,26]. Electronics and miniaturization play a key role in this process, allowing greater automation in industry and diffuse monitoring applications. The impact is increased by the expanding range of detectable parameters/analytes. Another pillar of progress concerns the continuous advances in transducer technologies tailored for specific applications and including optical and plasmonic, electrochemical, impedance, transistor-based and acoustic readout [27,28,29,30,31,32,33,34,35,36]. Among them, surface acoustic wave (SAW)-based devices have unique characteristics favoring high sensitivity.

Historically, SAW-based devices have been widely employed in the communication sector for their versatility and efficiency in controlling and processing electrical signals. In GHz information and communication technologies, SAW are largely used together with devices based on split ring resonators and ferromagnetic resonances within ferro/ferri-magnetic materials [37,38,39,40]. More recently, SAWs have begun to receive significant attention also in other fields and frontier research, from sensing and microfluidics to spintronics and quantum devices. In *“The 2019 surface acoustic waves roadmap”* [41], the state-of-the-art of SAW science and technology is summarized, discussing also future opportunities and challenges.

In the sensor field, SAW components are particularly suitable for developing ultrasensitive devices. Indeed, surface acoustic waves have an amplitude that decays exponentially away from the surface and exhibits a penetration depth of the order of the acoustic wavelength. Thus, such mechanical excitation is strongly localized in the surface region and consequently more sensitive to surrounding variations such as those associated with biorecognition events. This makes SAW transducers advantageous for biological and chemical sensing [41,42,43] and in detecting low-molecular-weight molecules [44]. Present applications of SAW sensors include on-field and wireless monitoring [45], operation in harsh environments [46], physical and chemical sensing including pressure, light, temperature and magnetic fields, as well as gases [14,47,48], pollutants [42], warfare agents [49], biomarkers and bacteria/cells detection [50,51,52].

In this work, we report on the parametric optimization of SAW delay lines on Lithium niobate for environmental monitoring applications. First, we evaluate the dependence of the transmitted signal on the device geometry, changing the main IDT (interdigital transducers) features such as the number of finger pairs, the finger overlap length and the distance between the emitter and the receiver. Then, the best-performing configuration was employed to implement SAW sensors for the detection of polystyrene particles simulating nanoparticulate/nanoplastics. Next, the SAW sensors were used for the first time to implement diagnostic tools able to detect Grapevine leafroll-associated virus 3 (GLRaV-3), one of the most widespread viruses in wine-growing areas [53]. The early detection of this virus by quick and cheap devices represents a very useful tool for combating this and other viruses of the vine, as these pathogens cannot be eliminated through conventional phytosanitary treatments, and specific regulations are in force in Europe for the marketing of plants free from this virus [54,55,56].

## 2. Materials and Methods

### 2.1. SAW Delay Lines Fabrication

**Figure 1:**
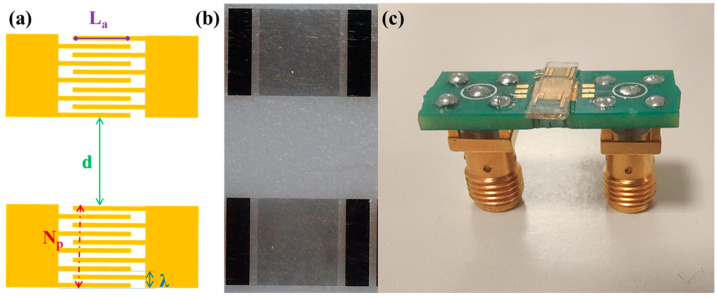
(**a**) Schematic of a SAW delay line illustrating the design features parametrically changed in the optimization study. (**b**) Optical microscope images of a SAW delay line and (**c**) a SAW device connected to a printed circuit board.

SAWs are typically generated on piezoelectric materials by the application of an oscillating potential to IDTs. Specifically, the IDTs consist of periodic metal electrodes whose periodicity set the SAW wavelength and determines the resonance frequency. Here, SAW delay lines consisting of two identical interdigital transducers were fabricated on Y-cut Lithium Niobate substrates (VM-TIM GmbH, Optomechanische Werke Victor-Goerttler-Str. 907745 Jena, Germany, 500 µm thick) by means of optical lithography (Figure 1). In detail, a 1.2 µm thick positive photoresist (AZ5214E) was spin-coated on a LiNbO_3_ substrate and exposed using a Karl Suss MA6 Mask Aligner. After the development, chromium and gold films, of 5 nm and 50 nm thickness, respectively were thermally evaporated and a lift-off step was performed.

### 2.2. SAW Delay Characterization

For characterization, each SAW device was successively connected to a PCB through Ø25μm aluminum wires using a microbonder. The amplitude and phase of the scattering parameter S_12_ (transmitted signal) were measured using a network analyzer (E5061B, Keysight, Santa Rosa, CA, USA).

### 2.3. Sensor Modification for Nanoparticulate Detection

Polystyrene beads of 200 nm diameter (Polybeads^®^, Polysciences Inc., Warrington, PA, USA) were used to simulate nanoparticulates/nanoplastics and evaluate the capability of the SAW sensors to detect them. The stock solutions received from the supplier had a 2.6% (w/v) beads concentration, corresponding to 0.026 g/mL in aqueous solution. For our studies, 1 μL of this stock solution was diluted in 10 mL of water, resulting in a suspension having a 2.6 μg/mL beads concentration. Then, consecutive 0.5 μL drops of the diluted sample were deposited between the IDT transducers of the SAW devices to change the amount of adsorbed nanoplastics and test the SAW sensor response.

### 2.4. Sensor Functionalization for GLRaV-3 Detection

For biosensing applications, a 1 × 1 mm^2^ square made of chromium/gold was realized between the IDT transducers during lithography. Then, to enable GLRaV-3 detection in homogenized samples, this metallic square was functionalized with highly specific antibodies (the same as those employed in ref. [57]). Specifically, the functionalization process consisted of the following steps:

− overnight deposition of a mixed self-assembled monolayer (SAM) of mercaptoundecanoic acid (11-MUA) and 2-mercaptoethanol (2-ME) in a ratio of 1:5 (0.2 mM of 11-MUA and 1 mM of 2-ME) in ethanol;
− 30 min incubation with N-hydroxysuccinimide (NHS, 0.05M) and N-ethyl-N-(3-di-methylaminopropyl) carbodiimide hydrochloride (EDC, 0.2M) in ultra-pure water to achieve activation of the COOH groups and form reactive N-hydroxysuccinimide esters;
− two hours incubation with Protein G (50mg/L) in PBS solution;
− 20 min incubation in an ethanolamine solution (1 M) in ultra-pure water;
− 15 min passivation with Bovine Serum Albumin (1 mg/mL) in PBS pH = 7 to saturate residual free electrode sites.
− one hour incubation with Agritest GLRaV-3 antibodies, diluted 1:1000 in PBS.

All the functionalization steps are realized at room temperature. All reagents except the antibodies were purchased from Sigma Aldrich Burlington.

Once the sensing device has been functionalized, it was incubated with serial dilutions of GLRaV-3 samples (1:10, 1:20, 1:50) for 1 h at room temperature and subsequently washed with Milli-Q water.

## 3. Results and Discussion

### 3.1. Sensor Optimization

For the parametric optimization study, the responses of the realized devices were systematically compared while changing one design parameter at a time (see Figure 1 and Table 1). Specifically, the IDT wavelength λ was fixed at 16 μm and the metallization ratio was kept constant at 0.5, i.e., the same width (4 μm) for the fingers and spaces. We then changed other geometric parameters such as the number of finger pairs *N_p_*, the finger overlap length *L_a_*, the distance *d* between the emitter and the receiver, and the propagation direction (Table 1).

**Figure 2:**
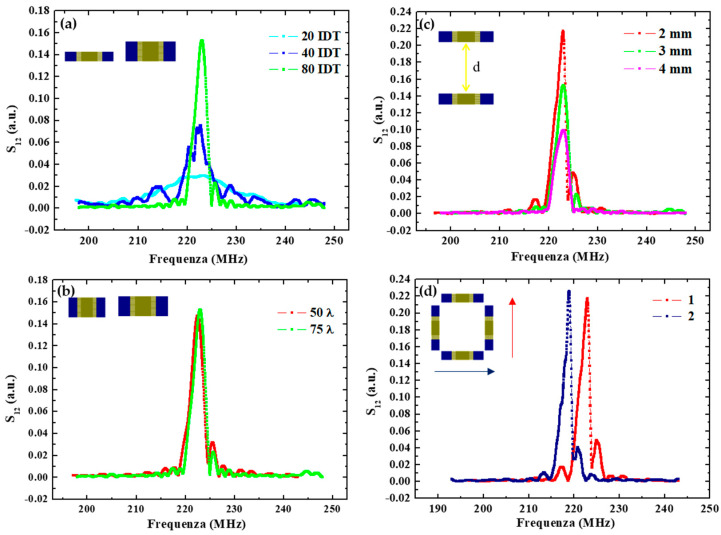
(**a**) Spectra comparison of SAW delay lines with different IDT pair numbers; (**b**) with different IDT overlap lengths; (**c**) with IDT transducers placed at different distances (d = 2 mm, 3 mm and 4 mm); (**d**) with different SAW direction propagation.

Concerning the finger parameters, Figure 2a compares the response of devices having an increasing number of pairs N_p_. The results indicate a corresponding increase in the delay line transmission at the resonance frequency moving from D1 to D2 and D3 with respectively 20, 40 and 80 IDT pairs. In addition, the bandwidth was observed to be proportional to 1/N_p_, going from about 12 MHz for devices with 20 IDT pairs, to ≈6 MHz and ≈3 MHz for IDT with 40 and 80 pairs, respectively.

Conversely, layouts with different finger overlap exhibit similar responses in terms of the transmitted signal, at least in the considered range of finger lengths. This is shown in Figure 2b in the case of the D4 and D6 devices having 910 and 1310 µm lengths, which correspond respectively to 50 λ and 75λ. No significant changes can be observed.

In Figure 2c, the responses of the D3, D4 and D5 devices realized by changing the IDT distance are reported. The transmitted signal at the resonance frequency is lower for a larger emitter-receiver distance. Specifically, it is reduced to about a half when increasing the distance from 2 mm to 4 mm as a result of the increasing energy losses. These energy losses are of dual nature: (1) an electrical loss due to SAW interaction with free charges in the substrate, and (2) a mechanical term due to viscosity and damping in the shear wave movements.

Finally, changing the propagation direction, the resonance peak moves toward a lower frequency—from 223 MHz to 219 MHz—as shown in Figure 2d, because of a lower acoustic wave velocity along this direction.

In conclusion, device D4 was selected as the best-performing for sensing applications, having the higher transmission and the sharpest resonance. These figures could be improved further by further increasing the number of pairs and reducing their distance. However, this would result in a more demanding lithography process (potentially reducing the fabrication yield) and a smaller space for nanoparticulate deposition, functionalization and analyte biorecognition (resulting in a lower adsorbed mass and induced change in the signal), respectively. Thus, we selected this layout as the preferred one for sensor implementation.

### 3.2. Application to Nanoparticulate Detection

Environmental monitoring is a primary global requirement for sustainable growth [14,58]. In this respect, aerosol particulate matter (PM) and nanoparticulates in particular represent a major threat, since exposure increases (for example) the risk for cancer and infertility [59,60,61,62,63,64,65]. Presently, airborne particulate levels are evaluated by tapered element oscillating microbalance spectrometers [66], ellipsometry [67] and light-scattering-based instruments [68], which have limitations for on-field monitoring campaigns and in detecting nanoparticles, which are the most dangerous. Thus, as a first application, the best-performing SAW layout resulting from the optimization study, D4, was employed for the detection of particles of sub-micrometer size opening the way to on-field nanoparticulate monitoring.

Specifically, in our experiments, the mass loading between the IDTs was monitored by measuring the transmitted signal S_12_ in its modulus and phase at the central frequency of the SAW delay lines resonance (223 MHz) before and after the deposition of each drop containing nanoparticles. In more detail, a 0.5 μL drop of the diluted sample containing the 200 nm Polystyrene beads (see Section 2.3) was deposited between the IDT transducers of the SAW devices and left to evaporate. The choice of a 0.5 μL volume (containing 1.3 ng beads) was made to have the drop entirely placed on the delay line path without spreading outside the sensing area. This is relevant in order to obtain a reproducible, quantitative measurement of the deposited particles. When the sample was completely dry, SAW spectra were acquired using a Keysight E5061B network analyzer. Then, a second drop was deposited to obtain a double amount of beads on the SAW path upon solvent evaporation. This process was repeated to increase the number of deposited nanoparticles and analyze the induced changes in the SAW signals.

**Figure 3:**
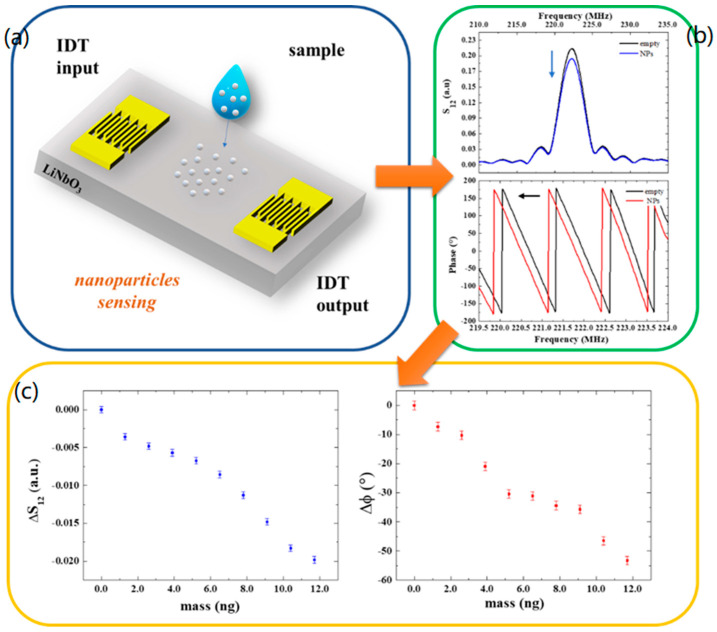
(**a**) Schematic illustration of a SAW device and nanoparticle deposition. (**b**) S_12_ and phase spectra of the SAW device without and with nanoparticles. (**c**) Transmitted signal variation and phase shifts as a function of the mass of particles at the resonant frequency.

The additional mass of nanoparticles deposited onto the sensor surface reduces the amplitude and the velocity of the wave producing an attenuation in the output signal and a shift in the phase toward lower values by a degree that is related to the mass of deposited nanoparticles (Figure 3a,b). Specifically, the phase exhibits a maximum variation of −53° after the deposition of nine drops, which corresponds to a mass of about 12 ng. In Figure 3c, the variation in the transmitted signal and phase shifts are reported as a function of mass as a response-dose calibration curve. The slope of the linear curve fitting the data points yields the mass sensor sensitivities:

$$S_{S12,\;SAW}\approx {\frac{\Delta S_{12}}{\Delta m}}_{SAW}=-0.002/ng\;S_{\varphi ,\;SAW}\approx {\frac{\Delta \varphi }{\Delta m}}_{SAW}=-4.3{}^{\circ}/ng$$

The detection limit (LOD) of the SAW sensors was estimated by dividing the amplitude *σ*_*S*12_ and phase uncertainties *σ_φ_* (calculated considering the average of residuals from the linear fits), respectively, with the sensitivity $S_{S12,\;SAW}$ and $S_{\varphi ,\;SAW}$, resulting in $LOD_{S_{12},SAW}$ = 0.5 ng and $LOD_{\varphi ,SAW}$ = 0.3 ng.

Table 2 shows a comparison of these values with those in the literature. The sensor sensitivity achieved in this work with delay lines realized on lithium niobate is better than the value obtained in our previous work in which the delay lines were realized on a quartz substrate. Moreover, the LOD value now obtained is lower than the case of delay lines in ref. [42] and comparable with the LOD value obtained with SAW resonators in ref. [69]. Thus, we conclude that the reported combination of piezoelectric material and layout improves the current state-of-the-art and opens the way to further (on-field) applications.

### 3.3. Application to GLRaV-3 Detection

Technologies for on-field monitoring of plant diseases have also attracted significant attention as detection tools are needed to combat the worldwide spread of phytopathological adversities and the consequent reduction in quantity and quality of yield and financial returns [16,72]. Thus, after polystyrene nanoparticles, we employed the optimized surface acoustic wave devices to evaluate their ability to detect GLRaV-3 particles as a second proof of application.

Among EU-regulated grapevine pathogens (Regulation (EU) 2016/2031), the GLRaV-3 is the most widespread virus, causing grapevine leafroll disease. The presence of the pathogen leads to the reddening or yellowing of leaves (in relation to red or white cultivars), dwarfism, and poor grape production [73]. The spread of the disease is caused by the propagation of infected plant material and insect vectors (several mealybugs are able to transmit the pathogen) [74]. Due to the lack of a cure (plant viruses cannot be eliminated through conventional agrochemical management), the use of virus-free stock and certification programs are established in several countries in order to produce, maintain, and commercialize healthy plants [73]. This virus can be considered paradigmatic as regards the monitoring of pathogens, as a) it is regulated at an international level, b) the absence of a cure makes monitoring essential to limit its spread, and c) it is particularly widespread in all viticultural areas of the world and has a significant economic impact.

**Figure 4:**
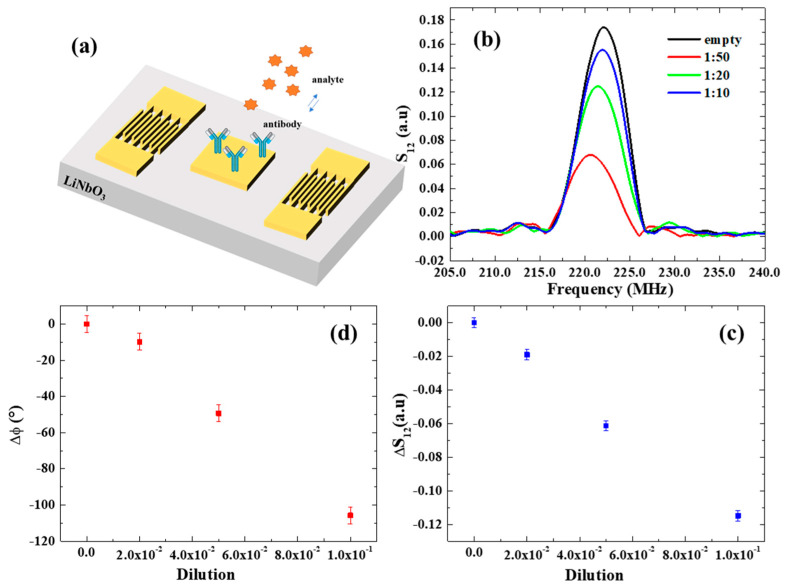
(**a**) Schematic illustration of a SAW device and electrode functionalization. (**b**) *S*_12_ spectrum in response to serial dilutions of GLRaV-3 virus source homogenate. (**c,d**) Transmitted signal variation and phase shifts as a function of dilution of GLRaV-3 virus source homogenate.

In this study, SAW assays were performed using dilutions of the original virus source (lyophilized GLRaV-3-infected woody tissues collected from naturally infected grapevine plants). Specifically, after functionalization with GLRaV-3 antibodies, the sensing device was incubated with serial dilutions of GLRaV-3 samples (1:10, 1:20, 1:50) for 1 h at room temperature, to allow the biorecognition with a volume of 0.5 µL (Figure 4a). Subsequently, the sample was washed with Milli-Q water to remove the excess, unbound antigen and dried with nitrogen flux. Afterward, SAW spectra were collected using the network analyzer for each dilution of GLRaV-3 samples in order to study the dependence of the SAW signals on the increasing virus concentration.

The resulting SAW spectra are shown in Figure 4b. A notable decrease in *S*_12_ and its phase φ was observed with increasing GLRaV-3 (i.e., at lower dilution ratios), providing a demonstration of the SAW device’s ability to monitor biorecognition events on its surface. The variations in the transmitted signal *S*_12_ and phase are reported as a function of dilution in Figure 4c,d and exhibit linear trends. The estimated mass sensor sensitivities are:

$$S_{S12,\;SAW}\approx {\frac{\Delta S_{12}}{\Delta m}}_{SAW}=-0.0013/ng\;S_{\varphi ,\;SAW}\approx {\frac{\Delta \varphi }{\Delta m}}_{SAW}=-2.9{}^{\circ}/ng$$

The detection limit (LOD) of our SAW sensors for the detection of GLRaV-3 virus was estimated by dividing the amplitude σ*_S12_ and phase uncertainties σ*_φ_ (calculated considering the average of residuals from the linear fits of *S*_12_ and phase), respectively, with the sensitivity $S^{*}{}_{S12,\;SAW}$ and $S^{*}{}_{\varphi ,\;SAW}$. In both cases, this results in a LOD of 1:500, which corresponds to a phase variation of Δφ = 1° and an associated mass variation of 0.2 ng, which is similar to the LOD estimated for nanoparticulate detection.

Notably, to date, there are no works in the literature that employ a SAW transduction mechanism for plant virus detection with which we can make a comparison. Until now this technology has only been used for the detection of human viruses, such as the Ebola Zaire virus [75], the papilloma virus [76], hepatitis B surface antibodies [77], and the influenza A virus [78] with LOD in a similar range. Compared to recently reported electrochemical impedance sensors for GLRaV-3, SAW transduction provides a remarkable five-times-better LOD.

## 4. Conclusions

In this work, a parametric optimization of SAW delay lines on LiNbO_3_ substrates was carried out. In particular, by modifying opportunely the delay line geometry—specifically, increasing the number of finger pairs and reducing the IDT distance—the transmitted signal was enhanced. Moreover, a variation of 4 MHz in resonant frequency was observed, changing the SAW direction of propagation. In this systematic study, we identified layout D4 as the best-performing for sensing applications, having the higher transmission and the sharpest resonance. This configuration was then employed to realize SAW sensors.

As aerosol particulate matter (PM) is a major threat, we first demonstrated the capability of the fabricated SAW devices for the detection of 200 nm polystyrene particles simulating nanoparticulates/nanoplastics. A mass sensor sensitivity of −4.3°/ng and a LOD of 0.3 ng were estimated, demonstrating a better performance than previous delay lines sensors, and comparable with the LOD achieved with SAW resonators thanks to the proposed combination of piezoelectric material and optimized layout.

Next, the SAW sensors were used for the first time to implement diagnostic tools able to detect Grapevine leafroll-associated virus 3 (GLRaV-3), one of the most widespread viruses in wine-growing areas. In this case, a LOD of around 1:500 was estimated from the noise level, applying a three-sigma criterion. Compared to recently reported electrochemical impedance sensors for GLRaV-3, SAW transduction provides a remarkable five-times-better LOD. This achievement opens the way to the application of miniaturized SAW sensors for the early detection of viruses and pathogens by quick and cheap devices providing a valuable means to fight the spreading of infectious diseases.

In conclusion, it is worth noting that portable readers for the operating frequency range of the optimized SAW sensors are commercially available. Thus, our two proofs of concept demonstrate the ability of miniaturized SAW sensors for carrying out on-field monitoring campaigns of nanoparticulates and plant pathogens with the potential (in the future) to replace the presently used heavy and expensive laboratory instrumentation, reducing the response time and overall costs.

## Figures and Tables

**Table 1:** Summary of the realized delay lines layouts.

Device	IDT Pairs N_p_	IDT Finger Length L(µm)	IDT Overlap Length L_a_(µm)	IDT Distance d (µm)	Direction
D1	20	1310	1200	3000	1
D2	40	1310	1200	3000	1
D3	80	1310	1200	3000	1
D4	80	1310	1200	2000	1
D5	80	1310	1200	4000	1
D6	80	910	800	2000	1
D7	80	1310	1200	2000	2

**Table 2:** Comparison of the literature work on the detection of nanoparticles by means of acoustic transduction methods with our results.

Transducer	Substrate	f_0_	Particle Material	Particle Diameter	Sensitivity $S_{m}$	Limit of Detection	References
Saw delay lines	LiNbO_3_	223 MHz	Polystyrene	0.2 µm	4.3 °/ng	0.3 ng	Present work
Saw delay lines	Quartz	206 MHz	Polystyrene	0.04 to 1 µm	0.4°/ng	1.9 ng	[42]
EIS	Glass		Polystyrene	0.04 to 1 µm	45 Ω/ng	2.8 ng	[42]
QCM	Quartz	4.988 MHz	Silicon dioxide	0.5 to 8 µm	0.2742 Hz/ng	3.65 ng	[70]
SAW resonators	Quartz	311.6 MHz	Polystyrene	2 µm	93.96 (Hz/min)/(ug/m^3^) with a flow rate of 13.5 mL/min	0.17 ng	[69]
SAW resonators	Quartz	262 MHz	Gold	750 nm	275 Hz/ng	0.21 ng	[71]

## Data Availability

Data are contained within the article.
